# Association of Long Versus Standard-Length Cephalomedullary Nail Constructs with Posterior Sagging in Intertrochanteric Fractures with Posteromedial Comminution: A Retrospective Comparative Study

**DOI:** 10.3390/jcm15187208

**Published:** 2026-09-17

**Authors:** Jun-Young Heu, Se-Won Lee, Ju-Yeong Kim

**Affiliations:** 1 Department of Orthopedic Surgery, Incheon St. Mary’s Hospital, College of Medicine, The Catholic University of Korea, 56 Dongsu-ro, Bupyeong-gu, Incheon 21431, Republic of Korea; med@live.co.kr; 2 Department of Orthopedic Surgery, Yeouido St. Mary’s Hospital, College of Medicine, The Catholic University of Korea, 10 63-ro, Yeongdeungpo-gu, Seoul 07345, Republic of Korea; 3 Department of Orthopedic Surgery, Gyeongsang National University Changwon Hospital, College of Medicine, Gyeongsang National University, 11 Samjeongja-ro, Seongsan-gu, Changwon 51472, Republic of Korea

**Keywords:** intertrochanteric fracture, cephalomedullary nail, posterior sagging, anteromedial cortical support, nail length, lesser trochanter

## Abstract

**Background:** Posterior sagging (PS) of the proximal fragment after cephalomedullary nailing (CMN) of intertrochanteric fractures leads to loss of anteromedial cortical support. **Methods:** We retrospectively reviewed 118 patients with AO/OTA 31-A2 intertrochanteric fractures (2007 classification). After an institutional protocol change, standard-length constructs with one distal screw were used until March 2019 (*n* = 65) and long constructs (≥300 mm) with two distal screws from June 2019 (*n* = 53); the cohorts were consecutive, not concurrent. **Results:** PS occurred in 23/118 patients (19.5%): 21/65 (32.3%) with standard-length versus 2/53 (3.8%) with long constructs (*p* < 0.001; adjusted odds ratio (OR) 0.08, 95% confidence interval (CI) 0.02–0.37). With standard-length constructs, PS was more frequent with big lesser trochanteric (LT) fragments (42.9% vs. 13.0%, *p* = 0.014); the interaction was not significant (*p* = 0.092). Long-construct use and a higher reduction score were associated with lower odds of PS, and older age and a big LT fragment with higher odds. Greater trochanter (GT) separation was present in 114 of 118 fractures and could not be evaluated as a risk factor. **Conclusions:** PS was associated with the standard-length construct and, within this group, with a big LT fragment. Nail length, distal locking, and treatment era could not be separated, so these findings show an association, not a causal effect of nail length. Functional outcomes were not assessed.

## 1. Introduction

Anteromedial cortical support is a key determinant of stability after cephalomedullary nail (CMN) fixation of pertrochanteric femur fractures [1,2]. Intraoperative techniques to obtain and verify anteromedial cortical support continue to be developed, reflecting its clinical importance [3]. Even when adequate cortical apposition is achieved intraoperatively, loss of reduction during follow-up is not uncommon, and re-displacement of the head–neck fragment converts an initially good or acceptable reduction into a poor one, predisposing to varus collapse, excessive sliding, and healing complications [2,4], which may ultimately require conversion arthroplasty [5].

One characteristic pattern of such loss of reduction is posterior sagging (PS) of the proximal fragment, in which the head–neck fragment translates posteriorly on the lateral radiograph with loss of anterior cortical contact despite adequate intraoperative reduction [6]. Kim et al. reported that PS occurred in 26% of patients after cephalomedullary nailing and was associated with separation of the greater trochanter (GT), which they proposed weakens posterior bony support around the proximal portion of the nail [6]. Patients with PS showed longer time to union, more nonunion, and worse ambulatory outcomes [6]. Separately, Song et al. described a “pendulum-like movement” of short nails within a wide medullary canal, in which the distal interlocking screw acts as a pivot and the proximal nail swings in the sagittal plane; a low filling ratio of the distal nail segment to the medullary canal in the lateral view was a significant risk factor for postoperative loss of anteromedial cortical support, with a threshold of 53% [7]. A three-dimensional computed tomography (CT) study of trochanteric fractures further showed that posterior coronal fragments involving the lesser trochanter (LT) and the posteromedial cortex are common in A2 fractures [8]. In such cases, lateral anchoring of the lag screw may not be achieved. A biomechanical study also showed that a lateral fracture line facilitates swing motion of the intramedullary nail [9]. Therefore, a standard-length straight nail is prone to sagittal swing.

**Figure 1:**
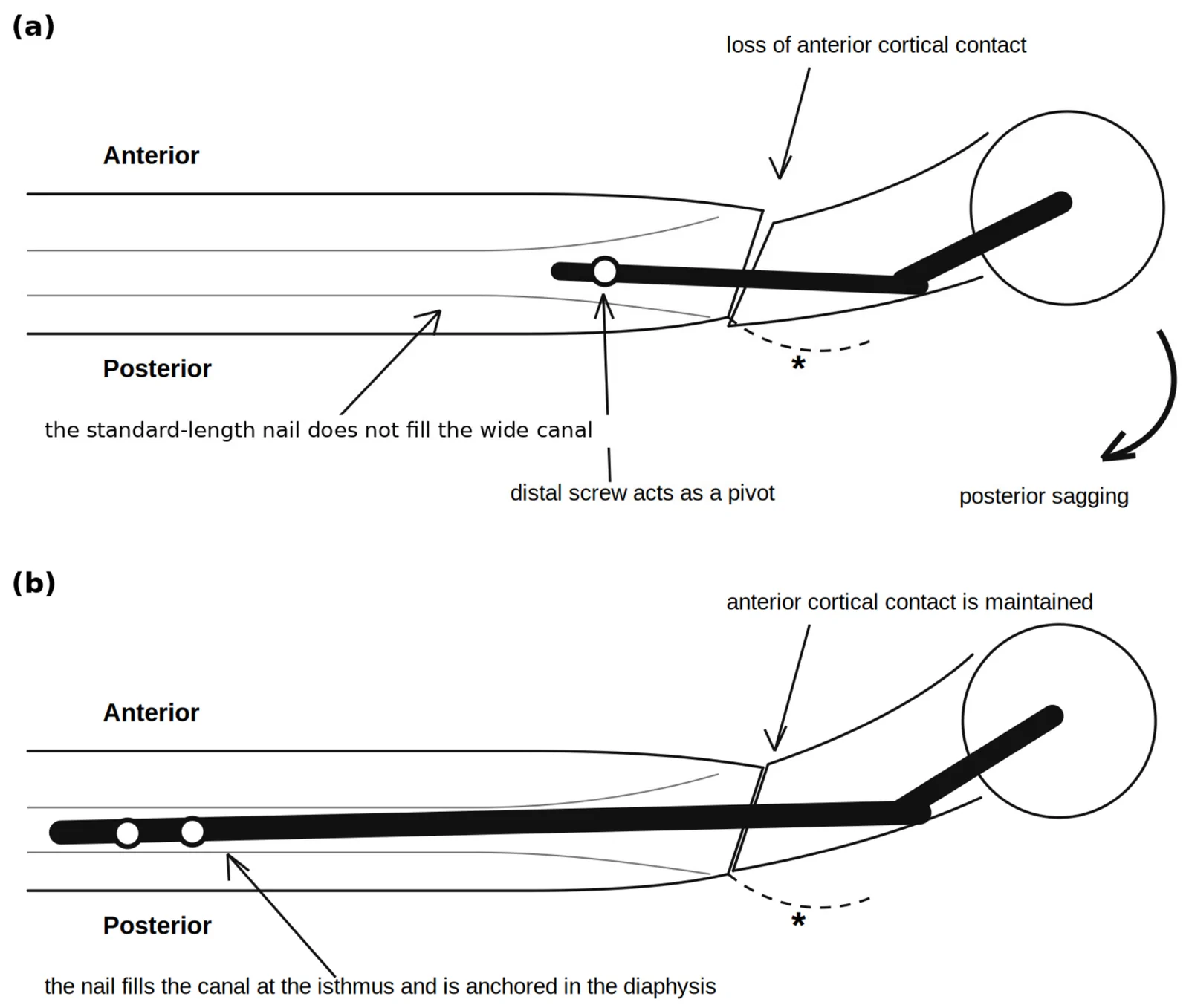
Schematic drawing of the proposed pendulum-like mechanism. (**a**) With a standard-length construct locked with one distal interlocking screw, the distal nail segment does not fill the wide medullary canal; the distal screw acts as a pivot, and the proximal nail and head–neck fragment can swing in the sagittal plane. (**b**) With a long construct that passes the isthmus and is locked with two distal interlocking screws, the distal nail fills the canal and is anchored in the diaphysis, and the sagittal swing is limited. The dashed lines indicate the fracture line and the posteromedial fragment including the lesser trochanter. The asterisk marks the posterior defect at the level of the lesser trochanter, where posterior support for the proximal fragment is lost.

In our experience, PS frequently occurs when posterior support for the proximal part of the CMN at the level of the LT is deficient, rather than in association with GT separation per se, and we have observed that long CMNs appeared to be associated with a lower incidence of PS even in fractures with large LT fragments. A long nail that passes the femoral isthmus fills the medullary canal distally and may act like a post anchored in the diaphysis and may suppress pendulum-like motion. This mechanism is a hypothesis of the authors and is illustrated in Figure 1. Although full-length nails were previously recommended by Chang et al. as a way to limit pendulum-like motion [10], and this was also noted by Song et al. [7], direct clinical comparisons of standard-length and long CMN constructs with respect to postoperative PS are lacking.

The primary objective of this study was to compare the incidence of PS between standard-length and long CMN constructs in AO/OTA 31-A2 intertrochanteric fractures with posteromedial comminution. The secondary objectives were to examine whether the size of the LT fragment and GT separation were associated with PS, and to compare radiographic and perioperative outcomes between the two constructs. We hypothesized that (1) long CMN constructs are associated with a lower occurrence of PS than standard-length constructs, and (2) with standard-length constructs, PS is more frequent when the LT fragment is large. Additionally, we examined whether GT separation, the previously reported risk factor [6], remained associated with PS when nail length and LT fragment size were taken into account.

## 2. Materials and Methods

### 2.1. Study Design and Patients

This was a retrospective comparative cohort study using two consecutive treatment eras defined by an institutional change in the CMN construct protocol. All patients with pertrochanteric fractures treated with CMNs by a single surgeon at a single university hospital between March 2017 and March 2022 were reviewed. Inclusion criteria were: (1) age ≥60 years; (2) fresh intertrochanteric femur fracture; (3) AO/OTA 31-A2 fracture, according to the original 2007 AO/OTA classification, with a fractured lesser trochanter and an intact lateral femoral wall; (4) availability of preoperative and intraoperative radiographs; and (5) postoperative radiographic follow-up of at least 3 months. Exclusion criteria were: (1) pathological fracture; (2) arthroplasty performed for the intertrochanteric fracture; (3) plate augmentation, including greater trochanteric reattachment devices and reconstruction plates; and (4) treatment during the transitional period of April and May 2019, when both constructs were in use (*n* = 5; three standard-length and two long). Eight otherwise eligible patients were excluded because radiographic follow-up was less than 3 months. Among these eight patients, two died within 3 months, and six were lost to follow-up. Four were treated in the standard-length era and four in the long-construct era. In total, 265 patients were screened; 134 did not meet the inclusion criteria, 5 were treated during the transitional period, and 8 had radiographic follow-up shorter than 3 months. A total of 118 patients met the criteria.

Fractures were classified according to the original 2007 AO/OTA classification [11]. In this classification, A2 indicates a multifragmentary pertrochanteric fracture with posteromedial comminution, and the subgroups A2.1 to A2.3 are divided by the number of intermediate fragments, regardless of the lateral wall thickness. In the 2018 revised classification [12], fractures with an intact lateral wall [13] (>20.5 mm) are placed in group A1.3, and only fractures with an incompetent lateral wall remain in group A2. Therefore, some fractures classified as A2.1 in the present study could be reclassified as A1.3 under the revised system, although the lateral wall thickness was not measured formally in this study. We used the original classification for two reasons. First, patient allocation and the nail-length protocol of our institution were based on the original classification during the whole study period. Second, our inclusion criteria already required an intact lateral wall.

The nail length was determined by a change in the institutional protocol, not by the surgeon’s preference within each period. Through March 2019, standard-length CMNs were used for A1 and A2 fractures, and long CMNs were used only for A3 fractures. From April 2019, the institutional protocol was changed, and long CMNs (≥300 mm) were used for A2 and A3 fractures. The five patients treated during the transitional period of April and May 2019 were excluded (exclusion criterion 4). The construct was therefore decided by the date of surgery, and the surgeon did not select between the two constructs case by case within each period. As a result, the standard-length cohort included the patients treated from March 2017 to March 2019 (*n* = 65), and the long-nail cohort included the patients treated from June 2019 to March 2022 (*n* = 53). According to the standard technique for each construct, all standard-length nails were fixed distally with one interlocking screw, and all long nails were fixed with two interlocking screws, without any exception. One hundred and ten patients were treated with the proximal femoral nail antirotation (PFNA; DePuy Synthes) and eight with the Gamma3 nail (Stryker). The PFNA-II was not used in this series. The nail diameter was selected for each patient according to the medullary canal, so a single diameter does not apply to either group, and this variation is reflected in the distal filling ratio. Except for the nail construct, the surgical technique, reduction methods, fracture table, imaging equipment, and postoperative rehabilitation protocol did not change during the study period. Patients started walking on the first postoperative day, and weight bearing as tolerated with a walker was allowed in all patients regardless of the construct. Perioperative transfusion followed the institutional threshold. Red-cell transfusion was given when the hemoglobin level was below 7 g/dL in patients without symptoms, and it was given at the discretion of the clinician in symptomatic patients with a hemoglobin level below 10 g/dL. This study was approved by the Institutional Review Board of our institution (approval No. SC22RISI0145; approved on 31 August 2022). The requirement for informed consent was waived because of the retrospective design.

### 2.2. Radiographic Assessment

**Figure 2:**
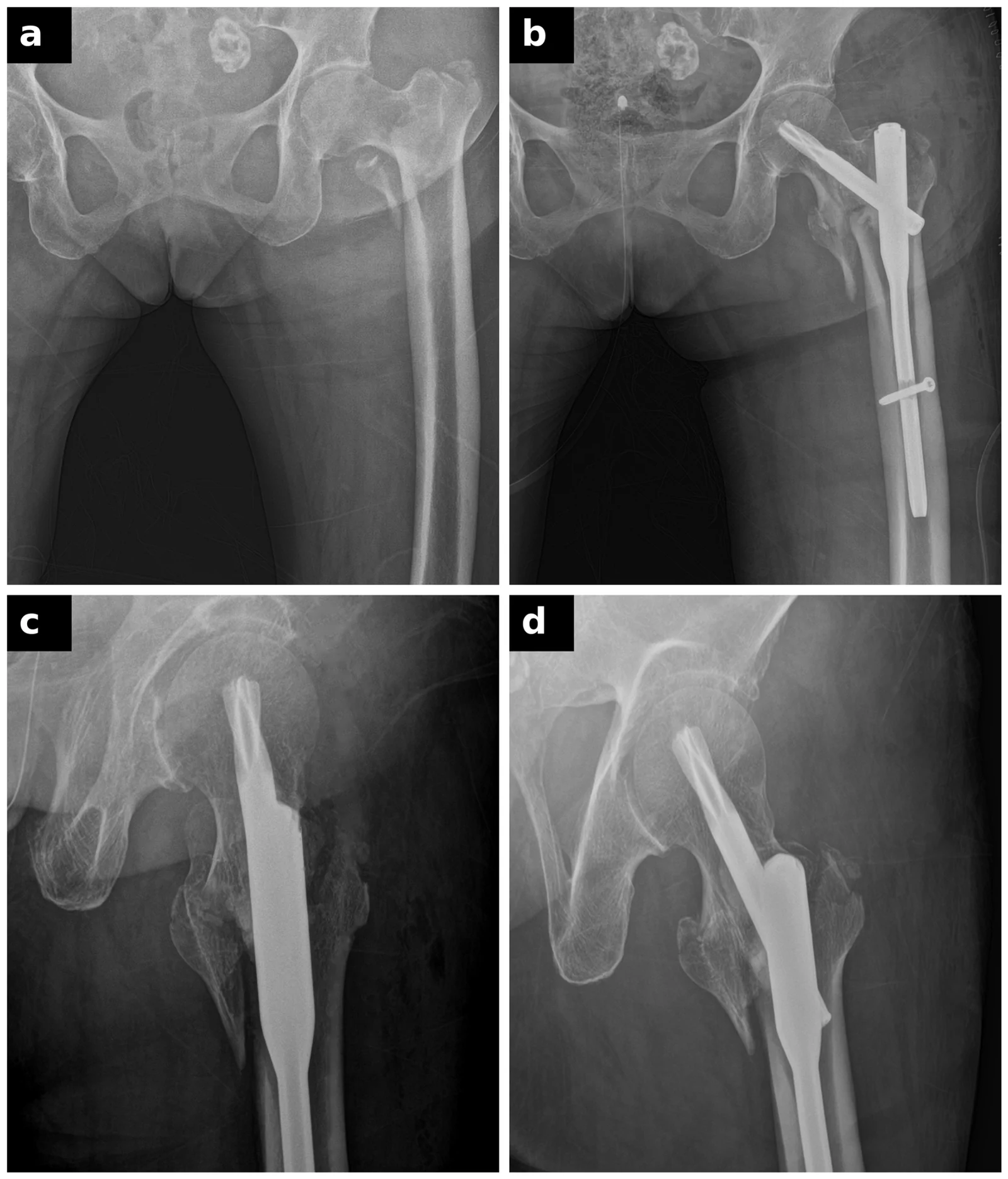
A 78-year-old woman with an AO/OTA 31-A2 fracture with a big lesser trochanteric fragment treated with a standard-length cephalomedullary nail. (**a**) Preoperative anteroposterior radiograph. (**b**) Immediate postoperative anteroposterior radiograph (day 0), showing the whole nail with one distal interlocking screw. (**c**) Immediate postoperative lateral radiograph (day 0), used as the baseline. (**d**) Lateral radiograph at the first outpatient visit (4 weeks after surgery): the proximal head–neck fragment has translated posteriorly around the nail with loss of anterior cortical contact (posterior sagging). Panels (**a**–**d**) are shown at the same magnification.

Posterior sagging was defined according to Kim et al. [6] as posterior translation of the proximal head–neck fragment with loss of anterior cortical contact at the fracture site on the lateral radiograph, when compared with the immediate postoperative lateral radiograph. Kim et al. applied this criterion on the translateral view; the projection differs, but the criterion is the same. Kim et al. referenced PS to the reduction before nail insertion, whereas our baseline was the immediate postoperative radiograph. Our definition therefore captures PS that develops after the immediate postoperative film, and posterior displacement that occurs during nail insertion is reflected in the reduction quality grade and is not counted as PS. The term posterior sagging has also been used by Lim et al. [14] for a preoperative posterior displacement of the distal shaft fragment, which is a different state in the opposite direction. Kuroda et al. [15] describe anterior redisplacement of the distal fragment after nailing; this is the same movement as posterior sagging of the head–neck fragment, described from the shaft side. In this study, PS means posterior translation of the proximal head–neck fragment relative to the shaft after the immediate postoperative film, following Kim et al. [6]. PS was evaluated on the first outpatient follow-up radiograph, which was taken 2 to 6 weeks after surgery. The lateral radiographs were standard lateral hip radiographs obtained by the radiology department according to its routine protocol, which was used at every assessment point and did not change during the study period. Radiographs were obtained according to the routine clinical schedule of our department, about every 6 to 7 days during admission and monthly until 3 months after surgery; this schedule was a clinical routine, not a study protocol. PS was determined on the first outpatient lateral radiograph only, and later films were used to confirm that no additional PS appeared. All images used for the assessment were those of the first outpatient visit in all 118 patients. Two orthopedic surgeons (J.-Y.H. and S.-W.L.) independently judged the presence of PS without knowing the clinical information. The cases were reviewed in random order, and the observers did not know the exact follow-up interval. Blinding to the CMN construct was not possible because the implant is visible on the radiograph. The inter-observer agreement was substantial (Cohen’s κ = 0.775), and disagreement occurred in 9 of 118 cases, which were resolved by consensus. To support the reproducibility of this qualitative judgment, we also measured, on the same view and after correction of magnification, the distance (mm) between the anterior cortical line of the proximal head–neck fragment and the anterior cortical line of the distal fragment, perpendicular to the anterior cortex of the distal fragment at the fracture site, and the anterior cortical thickness of the distal fragment at the same level. An operational quantitative criterion was applied, in which displacement equal to or greater than one anterior cortical thickness was regarded as PS. The agreement between the qualitative consensus judgment and this quantitative criterion was 113 of 118 cases (95.8%). In all analyses, the qualitative consensus judgment was used as the primary definition (Figure 2).

The quality of fracture reduction was graded on the immediate postoperative images using the 4-point criteria of Chang et al. [1], and the score cutoffs were applied as described by Song et al. [7]. One point was given for each of the following: (1) normal or slightly valgus alignment on the anteroposterior (AP) view, (2) angulation of less than 20 degrees on the lateral view, (3) positive or neutral medial cortical apposition, and (4) smooth anterior cortical apposition on the lateral view. Scores of 4, 3, and 2 or less were classified as good, acceptable, and poor, respectively.

**Figure 3:**
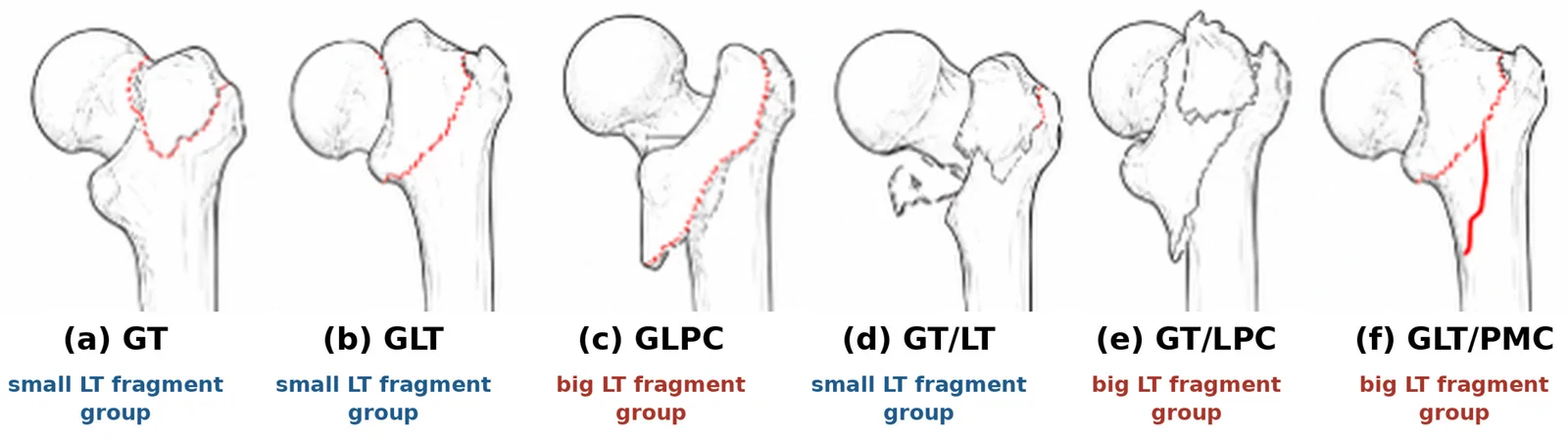
Schematic illustrations of representative coronal fragment patterns and their classification by LT fragment size, drawn based on the patterns described by Cho et al. [8]. The pattern name and the LT fragment group are given under each panel: (**a**) GT, (**b**) GLT, (**c**) GLPC, (**d**) GT/LT, (**e**) GT/LPC, and (**f**) GLT/PMC. The GLPC, GT/LPC, and GLT/PMC patterns form the big LT fragment group, and the GT, GLT, and GT/LT patterns form the small LT fragment group, according to the size rule described in the Methods. GLPC, greater trochanter–lesser trochanter–posteromedial cortex fragment; GLT, greater trochanter and lesser trochanter fragment; GT, greater trochanter fragment; LPC, lesser trochanter and posteromedial cortex fragment; PMC, posteromedial cortex; LT, lesser trochanter. This grouping is an operational classification of the present study and is not part of the original classification [8].

Preoperative CT, including three-dimensional reconstruction, was routinely obtained in all patients. Therefore, fracture morphology was assessed using both plain radiographs and preoperative 3D CT images. Coronal fragment patterns were named according to Cho et al. [8]: GT, GLT, and GLPC (single fragments) and GT/LT, GT/LPC, and GLT/PMC (two fragments). In the whole cohort (standard-length/long), the patterns were GT in 11 (5/6), GLT in 18 (11/7), GLPC in 40 (24/16), GT/LT in 7 (5/2), GT/LPC in 22 (9/13), and GLT/PMC in 16 (9/7) patients, and 4 (2/2) patients had no coronal fragment. The LT fragment size was classified at the original reading of the preoperative radiographs and 3D CT reconstructions. Before the reading, we set the rule that a posteromedial fragment larger than about twice the lesser trochanteric prominence would be regarded as a big LT fragment. This is an arbitrary rule of the present study. It was applied by visual comparison of the greatest dimension of the posteromedial fragment with the lesser trochanteric prominence on the posterior and medial views of the 3D reconstruction, without measurement. The GLPC, GT/LPC, and GLT/PMC patterns satisfied this rule and formed the big LT fragment group; the GT, GLT, and GT/LT patterns and fractures without a coronal fragment formed the small LT fragment group (Figure 3). GT separation was defined as the presence of a separate greater trochanteric coronal fragment according to Kim et al. [6]; it is therefore present in all patterns except the fractures without a coronal fragment. During the second revision, we found that the pattern originally labelled LPC in our records corresponds to GT/LPC and that GT separation had been coded as absent in these 22 patients; this coding error was corrected, and all analyses involving GT separation were repeated. Coronal fragment patterns were independently assessed by two orthopedic surgeons, with disagreements resolved by consensus. Before consensus, the inter-observer agreement at the original reading was κ = 0.91 (95% confidence interval [CI] 0.84–0.98) for the LT fragment size classification and κ = 0.90 (95% CI 0.83–0.97) for GT separation; the coding correction described above was made at the database level and did not involve re-reading of the images.

The tip–apex distance (TAD) was measured on the immediate postoperative AP and lateral radiographs according to the method of Baumgaertner et al. [16]. The magnification was corrected using the known diameter of the nail. The filling ratio of the distal nail segment to the medullary canal was measured on the immediate postoperative AP and lateral views, as described by Song et al. [7]. The neck–shaft angle (NSA) was measured on the AP views of the immediate postoperative and final follow-up radiographs. Blade sliding was defined as the change in the lateral protrusion of the helical blade between the immediate postoperative and final radiographs. Cut-out was checked on the follow-up radiographs. Nonunion was evaluated only in the patients who were followed up for at least 9 months (*n* = 81).

### 2.3. Clinical Data

Operative time and estimated blood loss were obtained from the anesthetic records. Perioperative red-cell transfusion (any versus none during the perioperative period) was obtained from the medical records.

### 2.4. Statistical Analysis

Continuous variables are presented as mean ± standard deviation, and they were compared using Welch’s t-test. Categorical variables were compared using the Pearson χ^2^ test, and Fisher’s exact test was used when the expected counts were small. For the comparison of the three levels of reduction quality between the nail-length groups, an exact (Monte Carlo) test was used. The number of PS events (*n* = 23) was small compared with the number of covariates. Therefore, Firth’s penalized-likelihood logistic regression was used for the multivariable analysis. Age, sex, side, LT fragment size, reduction score (entered as a continuous variable; 2 = poor, 3 = acceptable, 4 = good), and CMN construct (long vs. standard-length) were entered into the model at the same time. GT separation, the previously reported risk factor [6], was not entered because, after correction of the coding, it was present in 114 of 118 fractures (Section 2.2). The other covariates were selected a priori on clinical grounds: age, sex, and side as basic characteristics, LT fragment size as the study hypothesis, reduction score as the established predictor of loss of reduction [1,7], and the CMN construct as the exposure of interest. Data-driven variable selection was not performed. The distal filling ratio was not entered into the model. It is largely determined by the nail length and diameter, so it is an intermediate variable between the construct and PS. If it is adjusted for, part of the association under study is removed. Confidence intervals were calculated as Wald-type intervals from the penalized fit, and *p*-values were obtained from the penalized likelihood-ratio tests. The following sensitivity analyses were performed: conventional maximum-likelihood logistic regression, analysis by treatment period for a temporal trend, analysis stratified by reduction quality, analysis restricted to PFNA cases, a parsimonious multivariable model, and an alternative coding of reduction quality as good versus non-good. A two-sided *p*-value of less than 0.05 was considered statistically significant. All analyses were performed with PASW Statistics for Windows, Version 18.0 (SPSS Inc., Chicago, IL, USA) and Python 3.12. Firth’s penalized-likelihood logistic regression and the penalized likelihood-ratio tests were performed with custom code in Python 3.12 (NumPy, SciPy) implementing Firth’s bias-reduced estimation with the Jeffreys prior. The temporal trend in PS incidence within the standard-length era was tested by logistic regression of PS on the date of surgery as a continuous variable (likelihood-ratio test). Other analyses were performed with SciPy and statsmodels.

## 3. Results

### 3.1. Patient Characteristics and Incidence of Posterior Sagging

PS occurred in 23 of 118 patients (19.5%). Patients with PS were older (83.9 ± 7.6 vs. 80.1 ± 8.3 years, *p* = 0.042). Sex, fracture side, TAD, and postoperative NSA did not differ between groups. Standard-length constructs were used in 91.3% of PS patients versus 46.3% of non-PS patients (*p* < 0.001), and both distal filling ratios were markedly lower in the PS group (lateral, 0.51 ± 0.08 vs. 0.73 ± 0.13; AP, 0.63 ± 0.07 vs. 0.81 ± 0.11; both *p* < 0.001). GT separation was present in 100% of PS patients and 95.8% of non-PS patients (*p* = 1.000). Worse reduction quality was strongly associated with PS (*p* < 0.001) (Table 1).

### 3.2. Posterior Sagging by CMN Construct (Long vs. Standard-Length), LT Fragment Size, and GT Separation

With standard-length CMNs, PS occurred in 21 of 65 patients (32.3%) and was significantly more frequent with big LT fragments (18/42, 42.9%) than with small LT fragments (3/23, 13.0%; *p* = 0.014). With long CMNs, PS occurred in only 2 of 53 patients (3.8%), with no difference by LT fragment size (2.8% vs. 5.9%, *p* = 0.543). GT separation was present in 114 of 118 fractures (96.6%); PS occurred in 23 of 114 (20.2%) with and in 0 of 4 without GT separation (*p* = 1.000), so its association with PS could not be evaluated (Table 2). The CMN construct × LT fragment size interaction term was not statistically significant (Firth likelihood-ratio *p* = 0.092).

### 3.3. Multivariable Analysis

On Firth penalized logistic regression, long CMN use (adjusted odds ratio [OR] 0.08, 95% CI 0.02–0.37, *p* < 0.001), big LT fragment (OR 4.25, 95% CI 1.07–16.86, *p* = 0.028), higher reduction score (OR 0.11 per 1-point increase, 95% CI 0.04–0.33, *p* < 0.001), and older age (OR 1.11 per year, 95% CI 1.02–1.20, *p* = 0.005) were independently associated with PS. GT separation was not entered into the model because it was present in 114 of 118 fractures. Conventional maximum-likelihood estimates were similar (long CMN OR 0.05, *p* = 0.001) (Table 3). Because the number of events was small, especially in the long-construct group, these multivariable results should be regarded as adjusted exploratory estimates.

### 3.4. Comparison of the CMN Construct Cohorts

**Figure 4:**
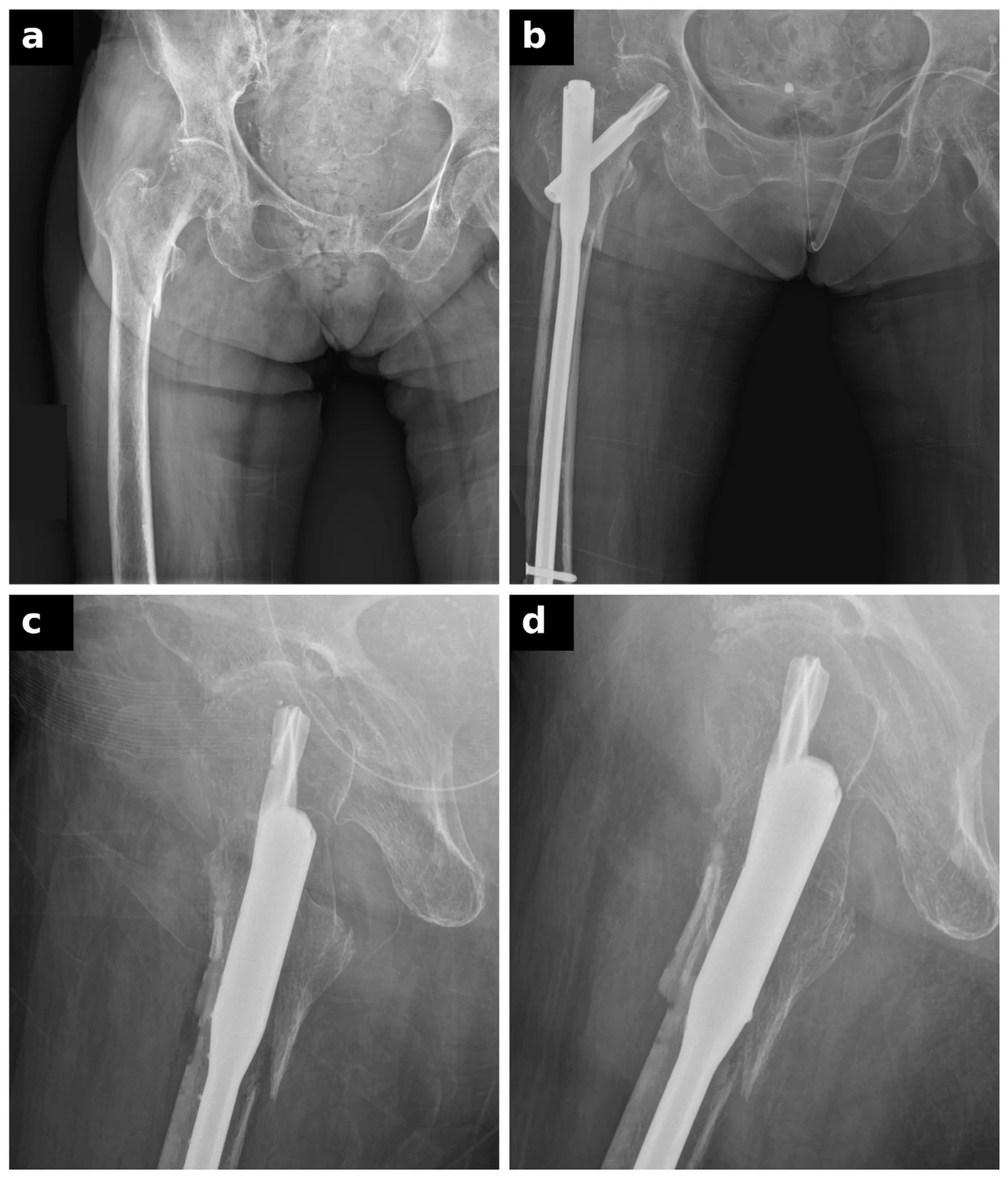
An 84-year-old woman with an AO/OTA 31-A2 fracture with a big lesser trochanteric fragment treated with a long cephalomedullary nail. (**a**) Preoperative anteroposterior radiograph. (**b**) Immediate postoperative anteroposterior radiograph (day 0), showing the whole nail passing the femoral isthmus with two distal interlocking screws. (**c**) Immediate postoperative lateral radiograph (day 0), used as the baseline. (**d**) Lateral radiograph at the first outpatient visit (4 weeks after surgery): anterior cortical contact is maintained without posterior sagging. Panels (**a**–**d**) are shown at the same magnification.

The two cohorts were comparable in age, sex, implant type, LT fragment size, GT separation, AO/OTA subtype distribution (*p* = 0.820), and follow-up duration. Reduction quality did not differ significantly (good, 52.3% vs. 71.7%; *p* = 0.093). This difference was not statistically significant, but it is clinically meaningful, so the two cohorts should not be regarded as fully comparable in this respect. The results stratified by reduction quality are given in Section 3.5. Owing to the implant design, both distal filling ratios were substantially higher with long CMNs (lateral, 0.84 ± 0.06 vs. 0.57 ± 0.07; *p* < 0.001); every long nail exceeded the previously reported 53% lateral threshold, whereas 27.7% of standard-length nails fell below it. Long CMNs required longer operative time (62.4 ± 10.5 vs. 52.7 ± 6.9 min, *p* < 0.001), greater estimated blood loss (143.7 ± 42.9 vs. 101.6 ± 29.7 mL, *p* < 0.001), and more frequent perioperative transfusion (60.4% vs. 41.5%, *p* = 0.042). The transfusion data are descriptive and unadjusted, because preoperative hemoglobin, anticoagulant use, and the number of transfused units were not analyzed. At final follow-up, varus drift of the NSA (1.9 ± 1.5° vs. 2.7 ± 2.2°, *p* = 0.030) and blade sliding (3.3 ± 1.6 vs. 4.3 ± 2.6 mm, *p* = 0.018) were smaller with long CMNs. Representative standard-length and long-construct cases are shown in Figure 2 and Figure 4, respectively. Cut-out (2 vs. 1) and nonunion among patients followed ≥9 months (2/45 vs. 1/36) were rare in both groups (Table 4). These events were uncommon, and the study was not powered for them. Therefore, the absence of a significant difference should not be interpreted as evidence that the two constructs have equal rates of these complications.

### 3.5. Sensitivity Analyses

Within the standard-length era, there was no evidence of a temporal decline in PS incidence (8/28 [28.6%] in 2017, 11/31 [35.5%] in 2018, and 2/6 [33.3%] in early 2019; *p* for temporal trend = 0.722), arguing against a gradual learning-curve effect as the primary explanation for the between-cohort difference. The association persisted within strata of reduction quality (good reduction: 0/38 [0%] with long vs. 5/34 [14.7%] with standard-length constructs; acceptable reduction: 1/14 [7.1%] vs. 14/29 [48.3%]) and after excluding the three patients with poor reduction (1/52 [1.9%] vs. 19/63 [30.2%]) (Table 5). When the analysis was restricted to PFNA cases, PS occurred in 2/50 (4.0%) with long versus 21/60 (35.0%) with standard-length constructs (*p* < 0.001). In a parsimonious Firth model including only nail construct, age, LT fragment size, and reduction score, the association of the long construct with PS was essentially unchanged (adjusted OR 0.07, 95% CI 0.02–0.36, *p* < 0.001), as it was when reduction quality was modeled as good versus non-good (adjusted OR 0.10, 95% CI 0.02–0.41, *p* < 0.001). Finally, within the standard-length group alone, the lateral distal filling ratio was lower in patients who developed PS than in those who did not (0.49 ± 0.04 vs. 0.60 ± 0.04, *p* < 0.001), supporting the proposed mechanism independently of the between-construct comparison.

## 4. Discussion

The main finding of this study is that long CMN constructs (≥300 mm, locked with two distal interlocking screws) were associated with a markedly lower occurrence of postoperative PS than standard-length constructs in AO/OTA 31-A2 intertrochanteric fractures (3.8% vs. 32.3%), and that this association remained strong in the adjusted analysis (adjusted OR 0.08, 95% CI 0.02–0.37) after adjustment for age, sex, side, reduction quality, and LT fragment size, and was robust across sensitivity analyses addressing temporal trends, reduction quality, model specification, and implant type. In fractures with a big posteromedial LT fragment treated with standard-length constructs, nearly half developed PS. To our knowledge, this is the first clinical study that directly compares a standard-length construct with one distal interlocking screw and a long construct with two distal interlocking screws with respect to PS of the head–neck fragment.

Our findings extend, and partly revise, the existing understanding of the mechanism of PS. Kim et al. attributed PS to GT separation weakening posterior support around the proximal nail [6]. In our cohort, restricted to A2 fractures, in which coronal fragments are very common, GT separation was present in 97% of patients (114 of 118) after correction of the coding described in Section 2.2. Because only four fractures were GT separation-negative, its association with PS could not be evaluated reliably, and GT separation was therefore not entered into the multivariable model. Our data therefore neither support nor refute the mechanism proposed by Kim et al. [6]. Among the fracture- and construct-related variables of interest, LT fragment size and the nail construct were associated with PS. We interpret these findings within the “pendulum-like movement” framework of Song et al. [7]: when the posteromedial buttress at the level of the LT is deficient, and the distal nail incompletely fills a wide canal, the distal interlocking screw acts as a pivot around which the proximal nail and head–neck fragment swing posteriorly in the sagittal plane. A long CMN construct that passes the isthmus may act like a post anchored in the diaphysis; in addition, the second distal interlocking screw used with long nails provides a longer fixation base that may itself resist sagittal swing, as suggested by Song et al. [7]. Consistent with this mechanism, the lateral distal filling ratio in our series was 0.84 with long versus 0.57 with standard-length constructs, all long CMN constructs exceeded the 53% threshold reported to be associated with a lower risk of loss of anteromedial cortical support [7], the filling ratio was markedly lower in patients who developed PS, and—within the standard-length group alone—patients who developed PS had lower filling ratios than those who did not (0.49 vs. 0.60). However, the filling ratio in this study should be regarded as a construct-level variable that is consistent with the proposed mechanism, rather than a formally tested mediator. After correction of the coding (Section 2.2), GT separation was present in 114 of 118 fractures of the present cohort, which included only A2 fractures with posteromedial comminution and an intact lateral wall, so its association with PS could not be evaluated. This does not exclude a contribution of GT separation in other fracture patterns, and our result should not be read as refuting the mechanism proposed by Kim et al. [6]. The difference from Kim et al. may also reflect the different baseline of the PS definition. Their incidence of 26% included PS that was already present on the immediate postoperative film, whereas our 19.5% counted only PS that developed after that film, so the two figures do not describe the same event. Two more points should be noted. First, the direction of the age association was opposite: their patients with PS were younger (72.7 vs. 78.5 years), whereas ours were older (83.9 vs. 80.1 years). The reason is not clear and may reflect differences in case mix and in the age structure of the cohorts, so the role of age in PS should be regarded as unsettled. Second, because the cohort of Kim et al. was treated with a PFNA locked with a single distal screw, it is comparable with our standard-length group, and their incidence of 26% is close to the 32.3% of our standard-length group.

Our findings should also be compared with the study of Kuroda et al. [15]. They analyzed anterior redisplacement of the distal fragment after nailing in 598 hips with AO/OTA A1–A3 fractures, which is the same event as PS in our study, described from the shaft side. They found no association with nail length (435 short nails <230 mm, 98 middle nails of 230–260 mm, and 65 long nails >260 mm) or with the canal-filling ratio, defined as the nail diameter divided by the canal diameter on the anteroposterior and oblique-lateral views. Instead, an anatomical reduction rather than a slightly posterior, buttressed reduction predicted redisplacement. Several differences may explain the discrepancy with our result: the small number of long nails in their series, the absence of stratification by the number of distal interlocking screws, the mixed fracture types, and a filling ratio that was defined differently from the distal-segment ratio of Song et al. [7] used here. The Chang score rewards anatomical apposition and does not record the direction of the sagittal reduction. Therefore, a gradual change toward a deliberately posterior, buttressed reduction during our study period could in principle mimic a construct effect. We could not test this possibility, because the direction of the sagittal reduction was not recorded in our data. Two findings do not support this explanation. PS incidence showed no temporal decline within the standard-length era (*p* for trend = 0.722), and the association between the construct and PS was maintained within each stratum of reduction quality, including the patients with good reduction.

The clinical relevance of PS is based on the established importance of anteromedial cortical support. Loss of anterior cortical contact permits uncontrolled impaction and has been linked to over-sliding, varus collapse, delayed union, and worse ambulatory recovery [2,6,7,9]. Consistent with this, in our series, higher reduction scores were associated with lower odds of PS, and the long-construct cohort showed smaller varus drift of the NSA and less blade sliding at final follow-up; these are supportive radiographic observations that are consistent with the main finding. PS in this study is a radiographic mechanical endpoint. Cut-out and nonunion were uncommon in both groups, and functional outcomes were not evaluated. Our data therefore show an association between the construct and a radiographic event. They do not show that long CMN constructs improve ambulation, pain, time to union, or reoperation.

These findings must be weighed against the critical points of long CMN constructs. In our series, long CMN constructs added approximately 10 min of operative time and 42 mL of estimated blood loss; a higher perioperative transfusion rate was also observed, although this finding may be influenced by unmeasured perioperative factors such as preoperative hemoglobin, anticoagulant use, and transfusion thresholds. The additional operative time in our series was smaller than the 18–22 additional minutes reported in comparative studies of short versus long cephalomedullary nailing [17,18,19,20,21,22], and this may reflect a standardized single-surgeon technique, whereas the additional blood loss was at the lower end of the reported range of about 40–140 mL. The evidence on later complications is split. Dunn et al. [17], Sadeghi et al. [18], and Cinque et al. [20] reported no difference in reoperation or peri-implant fracture, whereas Rajnish et al. [22] found more ipsilateral femoral shaft refractures with short nails (OR 1.60, 95% CI 1.14–2.24) and no difference in 30-day or 1-year mortality, and Tan et al. [21] suggested that long devices may have longer-term advantages. The apparently stronger association between fractures with a big LT fragment and PS is consistent with the proposed mechanism but should be considered hypothesis-generating, because the nail length × LT fragment size interaction was not statistically significant and the study was not powered to establish effect modification. Until this is confirmed, our data support further evaluation of long CMN constructs in A2 fractures with large posteromedial fragments, whereas for fractures with small LT fragments—in which PS occurred in 13% with standard-length constructs—the choice may reasonably be individualized. Our findings do not address A1 fractures, which were outside the inclusion criteria of this study. Fluoroscopy time and radiation dose, implant cost, implant removal, conversion arthroplasty, and peri-implant fracture were not recorded in our database, and anterior thigh pain was not assessed, so the overall balance of harm and benefit between the two constructs cannot be judged from our data. Protection of the entire femur against secondary peri-implant fracture is the classical argument for long nails, and this could not be evaluated in the present study. In frail elderly patients, the possible gain in early sagittal stability and the additional burden of the long construct should be weighed for each patient.

For current practice, our findings do not support a general recommendation to use long CMNs in all intertrochanteric fractures. In A2 fractures with posteromedial comminution, in which the posteromedial buttress is lost, the construct may be one of the factors that can be changed, together with the quality of reduction. Avoiding fixation failure is important, because the salvage operation is demanding. Failure of fixation of a proximal femoral fracture often requires conversion arthroplasty, and this procedure has a higher complication rate than primary arthroplasty [5]. Future studies should record nail length, the number of distal interlocking screws, the canal filling ratio, and the direction of the sagittal reduction as separate variables, because these factors were completely aligned in our cohorts and in most previous reports. Prospective multicenter comparison with a standardized radiographic protocol and structured longitudinal data collection is needed to confirm our findings.

### Limitations

This study has several limitations. First, and most importantly, nail length was determined by a protocol change, so the two cohorts are consecutive in time rather than concurrent: all standard-length nails were implanted in 2017–2019 and all long CMN constructs in 2019–2022. An era effect—most plausibly increasing surgeon experience or gradual changes in reduction technique—could therefore confound the association between nail length and PS. Several observations mitigate, although they cannot eliminate, this concern. The two cohorts were similar in demographics, fracture morphology (LT fragment size, GT separation), implant type, and follow-up duration; reduction quality, the most direct marker of surgical performance, did not differ significantly between periods (*p* = 0.093) and was adjusted for in the multivariable model, in which the association of long CMN constructs with PS remained strong (OR 0.08); the effect size (32.3% vs. 3.8%) is larger than would be expected from a learning curve in a surgeon who already had more than 10 years of hip-fracture practice at the start of the inclusion period; and the proposed mechanism is supported by an objectively measured intermediate variable (the distal filling ratio) that is a fixed property of the implant–canal construct rather than of the era; moreover, within the standard-length era itself, PS incidence showed no temporal decline (Section 3.5). Nevertheless, residual confounding by time cannot be excluded, and our results should be confirmed in concurrent or randomized comparisons.

Second, nail length was completely aligned with the distal locking configuration: all standard-length nails were locked with one distal interlocking screw and all long nails with two, according to the standard technique for each implant, without exception. The effects of nail length per se and of the additional distal interlocking screw therefore cannot be separated in our data; indeed, Song et al. suggested that two distal screws may themselves help prevent pendulum-like movement of the nail [7]. For this reason, we described the comparison throughout as a comparison between constructs, and the observed association should be attributed to the long CMN construct as implanted rather than to nail length in isolation.

Third, PS was assessed on the first outpatient follow-up radiograph obtained 2–6 weeks postoperatively, and imaging followed the clinical routine rather than a study protocol, so the exact interval varied between patients; late-onset PS could theoretically have been missed in patients imaged early. This risk is likely small, because in the only study with serial imaging, all PS developed within 2 weeks of surgery and none thereafter [6], and all of our patients had at least 3 months of follow-up imaging during which no additional PS was identified; still, we cannot fully exclude minor misclassification of PS timing. In addition, the intraoperative fluoroscopic images were not archived in a form that allows reliable re-scoring against the reduction before nail insertion. PS that occurred during nail insertion could therefore not be captured, and our incidence is not directly comparable with that of Kim et al. [6].

Fourth, radiographic interpretation is subject to observer variability. We addressed this for the primary outcome by using two independent observers with substantial agreement (κ = 0.775) and consensus adjudication, and by demonstrating 95.8% agreement with an operational quantitative criterion based on displacement relative to cortical thickness. Although observers were blinded to clinical information, blinding to the nail construct was inherently impossible because the implant is visible on radiographs, so detection bias cannot be fully excluded; the high agreement with the quantitative criterion mitigates, but does not eliminate, this concern. In addition, the continuous radiographic measurements (TAD, filling ratios, NSA, blade sliding) were performed by a single observer without formal intra- or inter-observer reliability testing, which we acknowledge as a limitation.

Fifth, GT separation was present in 114 of 118 fractures after correction of a coding error found during revision (Section 2.2). Its association with PS therefore could not be evaluated in this cohort, which included only A2 fractures with posteromedial comminution, and our data neither support nor refute the mechanism proposed by Kim et al. [6].

Sixth, the number of PS events (23) was modest relative to the number of covariates. We therefore used Firth penalized-likelihood regression, which is designed for such settings, and confirmed that conventional maximum-likelihood estimates were similar; the parsimonious and alternative-coding models in Section 3.5 also gave consistent results. Even so, some confidence intervals are wide, and the point estimates for estimates with wide confidence intervals should be interpreted with caution.

Seventh, because the reduction-quality score includes anterior cortical continuity, PS and reduction quality are conceptually related. We minimized circularity by grading reduction only on immediate postoperative images, which were obtained before PS determination; the inverse association between reduction score and subsequent PS can therefore be interpreted as prediction rather than tautology.

Eighth, fractures were classified according to the original 2007 AO/OTA classification rather than the 2018 revision. Because the 2018 revision incorporates lateral wall thickness [13] and reassigns lateral wall-intact fractures from A2.1 to A1.3, our cohort is not directly interchangeable with series defined by the 2018 system, and some fractures labeled A2.1 here might be classified as A1.3 under the revision, although lateral wall thickness was not formally measured. We consider this a matter of nomenclature rather than of case mix: all included fractures had posteromedial comminution with an intact lateral wall by explicit inclusion criteria, our primary comparison (nail length) and effect modifier (LT fragment size, derived from coronal fragment morphology) do not depend on the classification version, and the institutional nail-length protocol that generated the two cohorts was itself based on the original classification. Lateral wall thickness was not measured retrospectively, so formal reclassification under the 2018 system was not performed; readers should note that our results apply to lateral wall-intact pertrochanteric fractures with posteromedial comminution, regardless of the classification label.

Finally, this is a single-institution, single-surgeon study of a Korean population treated predominantly with one implant system, which may limit generalizability; bone mineral density was not available for all patients and was not modeled; functional outcomes and time to union were not systematically evaluated. Preoperative hemoglobin, anticoagulant use, and the institutional transfusion threshold were not modeled, so the higher transfusion rate in the long-construct group should be interpreted with caution. Eight otherwise eligible patients were excluded because of follow-up shorter than 3 months, so a small attrition bias cannot be excluded, although these patients were equally distributed between the two eras. In addition, the direction of the sagittal reduction was not recorded. A gradual change toward a deliberately posterior, buttressed reduction during the study period could in principle mimic a construct effect, and we cannot test this possibility with our data.

## 5. Conclusions

In this retrospective, sequential-cohort study of older patients with lateral wall-intact pertrochanteric fractures and posteromedial comminution, long cephalomedullary nail constructs with two distal interlocking screws were associated with a lower incidence of early posterior sagging, characterized by loss of anteromedial cortical support, than standard-length constructs with one distal screw. Clinical and functional outcomes were not evaluated. Within the standard-length group, a big lesser trochanteric fragment was associated with PS; GT separation was present in almost all fractures of this selected cohort and could not be evaluated. Because treatment era, nail length, canal filling, and distal locking configuration could not be separated, prospective concurrent studies are required before a causal or practice-changing conclusion can be drawn.

## Figures and Tables

**Table 1:** Characteristics according to posterior sagging.

	Total (*n* = 118)	PS (+) (*n* = 23)	PS (−) (*n* = 95)	*p* Value
Age, years	80.8 ± 8.3	83.9 ± 7.6	80.1 ± 8.3	0.042
Sex, male	33 (28.0%)	6 (26.1%)	27 (28.4%)	0.823
Fracture side, right	61 (51.7%)	12 (52.2%)	49 (51.6%)	0.959
CMN construct				<0.001
Standard-length construct	65 (55.1%)	21 (91.3%)	44 (46.3%)	
Long construct (≥300 mm)	53 (44.9%)	2 (8.7%)	51 (53.7%)	
LT fragment size, big	78 (66.1%)	19 (82.6%)	59 (62.1%)	0.062
GT separation	114 (96.6%)	23 (100%)	91 (95.8%)	1.000
Reduction quality				<0.001
Good	72 (61.0%)	5 (21.7%)	67 (70.5%)	
Acceptable	43 (36.4%)	15 (65.2%)	28 (29.5%)	
Poor	3 (2.5%)	3 (13.0%)	0 (0.0%)	
Tip–apex distance, mm	16.6 ± 1.7	17.2 ± 1.8	16.5 ± 1.7	0.123
Distal filling ratio, lateral	0.69 ± 0.15	0.51 ± 0.08	0.73 ± 0.13	<0.001
Distal filling ratio, AP	0.78 ± 0.12	0.63 ± 0.07	0.81 ± 0.11	<0.001
Postoperative NSA, °	130.0 ± 3.2	129.3 ± 3.5	130.1 ± 3.1	0.294

Values are mean ± standard deviation (SD) or *n* (column %). Welch’s t-test for continuous variables; Pearson χ^2^ (exact tests when sparse) for categorical variables. PS was determined on the lateral radiograph as loss of anterior cortical contact by consensus of two independent observers (κ = 0.775); agreement with the quantitative criterion (posterior displacement ≥ one anterior cortical thickness) was 113/118 (95.8%). GT separation was determined directly from the preoperative 3D CT images (definition of Kim et al. [6]). AP, anteroposterior; CMN, cephalomedullary nail; GT, greater trochanter; LT, lesser trochanter; NSA, neck–shaft angle; PS, posterior sagging.

**Table 2:** Posterior sagging by CMN construct, LT fragment size, GT separation, and reduction quality.

	Total	PS (+)	PS (−)	*p* Value
Standard-length construct	65	21 (32.3%)	44 (67.7%)	0.014 †
Small LT fragment	23	3 (13.0%)	20 (87.0%)	
Big LT fragment	42	18 (42.9%)	24 (57.1%)	
Long construct (≥300 mm)	53	2 (3.8%)	51 (96.2%)	0.543 †
Small LT fragment	17	1 (5.9%)	16 (94.1%)	
Big LT fragment	36	1 (2.8%)	35 (97.2%)	
GT separation	118	23 (19.5%)	95 (80.5%)	1.000
GT separation (+)	114	23 (20.2%)	91 (79.8%)	
GT separation (−)	4	0 (0%)	4 (100%)	
Reduction quality	118	23 (19.5%)	95 (80.5%)	<0.001
Good	72	5 (6.9%)	67 (93.1%)	
Acceptable	43	15 (34.9%)	28 (65.1%)	
Poor	3	3 (100.0%)	0 (0.0%)	

PS (+) and PS (−) are *n* (row %). † *p* values compare small vs. big LT fragments within each construct (Pearson χ^2^ for the standard-length construct; Fisher’s exact for the long construct); they do not compare the two constructs. The comparison between the two constructs is given in Table 1 (*p* < 0.001). The GT separation *p*-value compares PS incidence between GT (+) and GT (−) (Fisher’s exact test); reduction quality used an exact 3 × 2 test. The nail length × LT fragment size interaction was not significant (Firth likelihood-ratio *p* = 0.092). CMN, cephalomedullary nail; GT, greater trochanter; LT, lesser trochanter; PS, posterior sagging.

**Table 3:** Multivariable (Firth penalized) logistic regression for posterior sagging.

Variable	Adjusted OR	95% CI	*p* Value
Age (per year)	1.11	1.02–1.20	0.005
Male sex (vs. female)	0.99	0.27–3.60	0.987
Right side (vs. left)	0.79	0.24–2.57	0.693
Big LT fragment (vs. small)	4.25	1.07–16.86	0.028
Reduction score (per 1-point increase)	0.11	0.04–0.33	<0.001
Long construct (vs. standard-length)	0.08	0.02–0.37	<0.001

Firth’s penalized-likelihood logistic regression owing to the limited number of events (23) relative to covariates; all variables entered simultaneously. CIs are Wald-type from the penalized fit; *p*-values are penalized likelihood-ratio tests. Reduction score: 2 = poor, 3 = acceptable, 4 = good. GT separation was not entered because it was present in 114 of 118 fractures. Conventional maximum-likelihood estimates were similar (long construct OR 0.05, 95% CI 0.01–0.31, *p* = 0.001; big LT fragment OR 5.38, *p* = 0.028). CI, confidence interval; CMN, cephalomedullary nail; GT, greater trochanter; LT, lesser trochanter; OR, odds ratio.

**Table 4:** Characteristics and secondary outcomes according to CMN construct.

	Total (*n* = 118)	Standard (*n* = 65)	Long (*n* = 53)	*p* Value
Age, years	80.8 ± 8.3	80.3 ± 8.6	81.5 ± 7.9	0.432
Sex, male	33 (28.0%)	21 (32.3%)	12 (22.6%)	0.245
Implant, PFNA	110 (93.2%)	60 (92.3%)	50 (94.3%)	0.729
LT fragment size, big	78 (66.1%)	42 (64.6%)	36 (67.9%)	0.706
GT separation	114 (96.6%)	63 (96.9%)	51 (96.2%)	1.000
AO/OTA subtype (A2.1/A2.2/A2.3)	26/38/54	13/22/30	13/16/24	0.820
Distal interlocking screws	—	One	Two	—
Reduction quality (G/A/P)	72/43/3	34/29/2	38/14/1	0.093
Tip–apex distance, mm	16.6 ± 1.7	16.5 ± 1.6	16.8 ± 1.8	0.260
Distal filling ratio, lateral	0.69 ± 0.15	0.57 ± 0.07	0.84 ± 0.06	<0.001
Distal filling ratio, AP	0.78 ± 0.12	0.68 ± 0.07	0.89 ± 0.06	<0.001
Postoperative NSA, °	130.0 ± 3.2	129.7 ± 3.0	130.3 ± 3.4	0.386
NSA change at final FU, °	2.3 ± 1.9	2.7 ± 2.2	1.9 ± 1.5	0.030
Blade sliding, mm	3.8 ± 2.3	4.3 ± 2.6	3.3 ± 1.6	0.018
Operative time, min	57.0 ± 9.9	52.7 ± 6.9	62.4 ± 10.5	<0.001
Estimated blood loss, mL	120.5 ± 41.7	101.6 ± 29.7	143.7 ± 42.9	<0.001
Perioperative transfusion (any)	59 (50.0%)	27 (41.5%)	32 (60.4%)	0.042
Cut-out	3 (2.5%)	2 (3.1%)	1 (1.9%)	1.000
Nonunion (FU ≥ 9 mo, *n* = 81)	3/81 (3.7%)	2/45 (4.4%)	1/36 (2.8%)	1.000
Follow-up, months	13.8 ± 6.8	13.6 ± 6.4	13.9 ± 7.4	0.800

Values are mean ± standard deviation (SD) or *n* (column %). Welch’s t test for continuous variables; Pearson χ^2^ or Fisher’s exact test for categorical variables; reduction quality (G/A/P, good/acceptable/poor) used an exact (Monte Carlo) 3 × 2 test. Perioperative transfusion is any red-cell transfusion (recorded units of 0/1/2 dichotomized as any vs. none). NSA change = postoperative minus final follow-up NSA (positive = varus drift). Nonunion was assessed only in patients with ≥9 months of follow-up. Distal locking configuration was determined by the construct in all patients (one screw for standard-length, two for long nails). AP, anteroposterior; CMN, cephalomedullary nail; FU, follow-up; GT, greater trochanter; LT, lesser trochanter; NSA, neck–shaft angle; PFNA, proximal femoral nail antirotation.

**Table 5:** Posterior sagging according to the CMN construct within strata of reduction quality.

Reduction Quality	Standard-Length Construct	Long Construct
Good	5/34 (14.7%)	0/38 (0%)
Acceptable	14/29 (48.3%)	1/14 (7.1%)
Poor	2/2 (100%)	1/1 (100%)
Excluding poor reduction	19/63 (30.2%)	1/52 (1.9%)

Values are the number of patients with posterior sagging/the number of patients in that stratum (%). Reduction quality was graded on the immediate postoperative radiographs. CMN, cephalomedullary nail.

## Data Availability

The data presented in this study are available from the corresponding author upon reasonable request. The data are not publicly available due to privacy and ethical restrictions related to patient confidentiality.
